# Population genomics of an outbreak of the potato late blight pathogen, *Phytophthora infestans*, reveals both clonality and high genotypic diversity

**DOI:** 10.1111/mpp.12819

**Published:** 2019-05-30

**Authors:** Sundy Maurice, Melanie S. Montes, Bent J. Nielsen, Lars Bødker, Michael D. Martin, Carina G. Jønck, Rasmus Kjøller, Søren Rosendahl

**Affiliations:** ^1^ Section for Genetics and Evolutionary Biology, Department of Biosciences University of Oslo Blindernveien 31 Oslo 0316 Norway; ^2^ Department of Biology University of Copenhagen Universitetsparken 15 Copenhagen O 2100 Denmark; ^3^ Department of Agroecology Aarhus University Forsøgsvej 1 Slagelse 4200 Denmark; ^4^ Danish Centre for Food and Agriculture Aarhus University Blichers Allé 20 Tjele 8830 Denmark; ^5^ Centre for Geogenetics Natural History Museum of Denmark Sølvgade 83 Copenhagen‐K 1307 Denmark

**Keywords:** clonality, *Phytophthora infestans*, plant pathogen, ploidy, population structure, RAD sequencing, SNPs

## Abstract

An outbreak of the potato late blight pathogen *Phytophthora infestans* in Denmark was characterized in order to resolve the population structure and determine to what extent sexual reproduction was occurring. A standard set of microsatellite simple sequence repeats (SSRs) and single nucleotide polymorphism (SNP) markers generated using restriction site‐associated DNA sequencing (RAD‐seq) were employed in parallel. A total of 83 individuals, isolated from seven different potato fields in 2014, were analysed together with five Danish whole‐genome sequenced isolates, as well as two Mexican individuals used as an outgroup. From a filtered dataset of 55 288 SNPs, population genomics analyses revealed no sign of recombination, implying clonality. In spite of this, multilocus genotypes were unique to individual potato fields, with little evidence of gene flow between fields. Ploidy analysis performed on the SNPs dataset indicated that the majority of isolates were diploid. These contradictory results with clonality and high genotypic diversity may suggest that rare sexual events likely still contribute to the population. Comparison of the results generated by SSRs vs SNPs data indicated that large marker sets, generated by RAD‐seq, may be advised going forward, as it provides a higher level of genetic discrimination than SSRs.

## Introduction

In order to manage agricultural pathogens, it is crucial to understand the population structure underlying epidemics. The evolutionary potential of pathogens to overcome challenges such as plant defence and fungicide resistance is determined by the amount of standing variation in a population, mode of reproduction and gene flow between populations, and should therefore dictate the management strategies of said pathogens (McDonald and Linde, 2002). Pathogens with a mixed reproductive system are especially successful because asexual reproduction leads an accumulation of mutations between gametes, and every single sexual event is therefore expected to create a unique multilocus genotype, so that even extremely rare sexual events are sufficient to cause a high degree of variation in a population (Bengtsson, 2003). The pathogens best suited for invading new environments are therefore those with a very low level of sexual reproduction together with predominantly asexual reproduction (~95%) because of their high genetic variability and linkage disequilibrium, which may link a trait that is unfit in the source population to a trait that is fit in a new environment (Bazin *et al.*, 2014).

*Phytophthora infestans* (Mont. De Bary), the pathogen causing late blight on potatoes and tomatoes, is of large societal and economic importance, causing significant losses each year worldwide despite the very heavy use of chemical control. This has led to *P. infestans* being a well‐documented organism with several studies focused on the dispersal and population genetic structure of the plant pathogen. A system of 12 microsatellites, or  simple sequence repeats (SSRs), primers for *P. infestans* which are easily multiplexed, were developed by Lees *et al.* (2006) and Li *et al.* (2013a), and have since been widely implemented in studies worldwide, such as in Africa (Harbaoui *et al.*, 2014), Asia (Li *et al.*, 2013b), Europe (Chmielarz *et al.*, 2014), North America (Danies *et al.*, 2014) and South America (Delgado *et al.*, 2013).

In most parts of the world, including central Europe, studies based on SSRs have shown that a few clonal lineages usually dominate a region. In Scandinavia, however, studies have revealed a population structure that differs from other parts of Europe. Populations with highly diverse genotypes (Brurberg *et al.*, 2011; Montes *et al.*, 2016; Sjöholm *et al.*, 2013), the presence of two mating types (A1 and A2) in roughly equal proportion, and evidence of soil‐borne inoculum, oospores and over‐wintering in the soil have led to the general conclusion that sexual reproduction is taking place in Scandinavia (Yuen and Andersson, 2012). Genetic analyses for linkage disequilibrium and recombination based on SSR data have, however, led to conflicting results (Brurberg *et al.*, 2011; Montes *et al.*, 2016; Sjöholm *et al.*, 2013), so the degree to which sexual reproduction is actually occurring is still debated and would benefit from analysis with an alternative genetic marker system with higher resolution in detecting genetic variation at fine sampling scales.

In the past few decades, SSRs have been the molecular marker of choice for studying within‐species population‐level questions due to their rapid mutation rate and their relatively low price and ease of use once a system is developed for a given species (Jarne and Lagoda, 1996). However, it has proven difficult to find a mutation model that accurately describes the mutation process in SSR loci and incorporates the effects of homoplasy and the complex mutational biases involved (Amos, 2010, 2016; Jarne and Lagoda, 1996), rendering difficult any genetic analyses based on genetic distances. Additionally, SSRs are not evenly distributed throughout the genome, and their use as an estimator for genome‐wide diversity and heterozygosity is controversial (Ljungqvist *et al*., 2010; Väli *et al.*, 2008). Instead, single nucleotide polymorphisms (SNPs) provide an improved alternative for population genetic studies to conventional SSRs. SNPs have the added advantage of being more numerous and spread throughout the entire genome, including regions linked to genes (Defaveri *et al.*, 2013).

Restriction site‐associated DNA sequencing (RAD‐seq), in which only regions adjacent to restriction enzyme cutting sites are sequenced, greatly reduces the complexity of the genome and allows for higher throughput genotyping (Baird *et al.*, 2008). Through the selection of different or multiple restriction enzymes, RAD‐seq can be tailored according to the GC content in the genome or biological questions by predetermining the number of cut sites (Baird *et al.*, 2008). One question, however, is how efficient RAD‐seq can be for organisms with a high number of repetitive regions and a low level of polymorphism. *Phytophthora infestans* is an extreme example of such an organism. *Phytophthora infestans* has a genome of 240 Mbp, three to four times the size of closely related *P. sojae* and *P. ramorum*, of which about 74% is made up of highly repetitive regions; the remaining regions, in contrast, are highly conserved (Haas *et al.*, 2009). The density of single‐nucleotide variants in *P. infestans* is relatively low, at only 0.65/Kilobases (kb) on average between strains and 7.67/kb average with other species in its clade (Lamour *et al.*, 2012).

An additional limitation in early SSR studies of *P. infestans* is unclear ploidy state. Although a definitive method for counting the number of chromosomes in *P. infestans* still eludes researchers, the DNA contents of different strains have been characterized in a number of studies (Ritch and Daggett, 1995; Therrien *et al.*, 1989; Tooley and Therrien, 1987; Whittaker *et al.*, 1991). These studies have demonstrated that ploidy is variable between isolates, with diploid individuals from the Mexican centre of origin, while triploid, tetraploid or aneuploid individuals come from modern‐day Europe. For SSR‐based studies, this has led to some difficulties, particularly early on when most analytical software available was for handling diploid data, so that individuals with triploid SSR alleles were simply excluded from analyses. More recently, a method for determining the ploidy of *P. infestans* has been developed using the allele frequencies of biallelic SNPs using whole‐genome sequences (Yoshida *et al.*, 2013). To our knowledge, this method has yet to be implemented on sequences generated from a reduced‐representation sequencing such as RAD‐seq.

Therefore in this study, using SSRs and SNPs markers in parallel, we (i) reveal the population structure of an outbreak of *P. infestans*, (ii) examine the extent to which sexual reproduction is taking place and (iii) determine ploidy in a large number of modern *P. infestans* isolates from Denmark.

## Results

### Population genetic analyses

A negative inbreeding coefficient, *F*_IS_, may indicate a clonal population structure because of an excess of observed heterozygotes compared to expected heterozygotes. The overall *F*_IS_ was −0.10 and −0.61 calculated from SSR and SNP datasets, respectively. In addition, the *F*_IS_ estimated from SNPs was also negative within each population (Table 1). When the expected heterozygosity was plotted against the observed, the observed heterozygosity was larger than the expected (Fig. S1). The overall *F*_ST_ based on SSR markers was 0.065 and was found to indicate significant population structure (*P* = 0.002). Likewise, we obtained a low but significant *F*_ST_ based on SNP markers (*F*_ST_ 0.0025, *P* < 0.001). Pairwise *F*_ST_ values between populations are listed in Table S3 **.** Tajima's *D* values were positive for all sampling fields tested in the current study, indicating balancing selection or a population bottleneck (Table 1).

**Table 1:** Genetic parameters estimated from SNPs data.

Population ID	SNPs				
	*N*	*F*_IS_	*H*_S_	Tajima's *D*	SD
M3	2	−0.655	0.262	1.328	0.797
M4	16	−0.476	0.256	1.234	1.021
M5	18	−0.471	0.254	1.231	1.052
M6	5	−0.579	0.255	1.196	0.793
M7	8	−0.513	0.257	1.154	0.906

Inbreeding coefficient (*F*_IS_), gene diversity (*H*_S_) and mean Tajima's *D* estimates with standard deviation (SD) were calculated per field for 2014 population with number of individuals (*N*). Genetic estimates were generated from 8875 SNPs with a maximum of 25% missing data at each locus. Tajima's *D* was calculated across 50 kb window.

**Figure 1:**
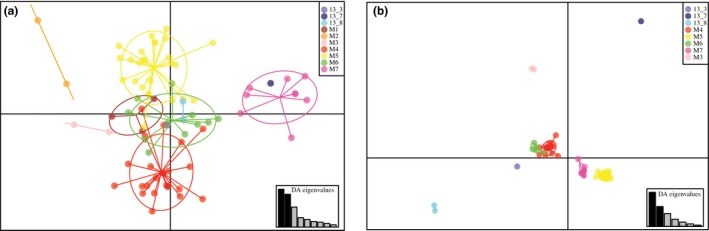
DAPC computed using ten SSRs (a) and 8875 SNPs (b) in 2013 and 2014 populations of *Phytophthora infestans* collected in seven fields (M1–M7) in Denmark. Scatterplots represent the distribution of individuals (dots) and fields (colour) as inertia ellipses, with first 21 and ten PCs retained for plots a and b, respectively. Samples from 2013 are represented in shades of blue, while those isolated in 2014 (M) are in shades of red. The insets indicate the eigenvalues of the DAPC analysis, with dark bars representing axis 1 and 2 of the plots.

Discriminant analysis of principle components (DAPC) analyses performed on both the SSR and SNP datasets showed a clear pattern of grouping according to the field of origin (Fig. 1), suggesting strong population structure. Clustering performed better with the 8875 SNPs than with SSR makers, with higher membership scores for the defined clusters. Though there was no significant signal of isolation by distance based on a Mantel test with 1000 permutations for either dataset (SSR *P* = 0.511, SNP *P* = 0.114), closely located fields were separated by a short distance in the DAPC. The grouping of fields along the axes was not correlated to potato varieties. When the 2013 and 2014 populations were compared (Fig. S5), analysis of the posterior probabilities showed that the proportion of successful reassignment of individuals to their population of origin ranged from 0.5 (field M3) to 1.0 (all 2013 individuals, Mexican outgroup and field M6), 0.875 overall, and revealed some admixture between populations. A tree based on bitwise genetic distance did not show any strong patterns of grouping by sampling field, except for the clear separation of the Mexican outgroup individuals and the strong pairing of individuals to their biological and technical replicates. However, bootstrap support was generally low or non‐existent (Fig. S2).

### Sexual recombination

**Figure 2:**
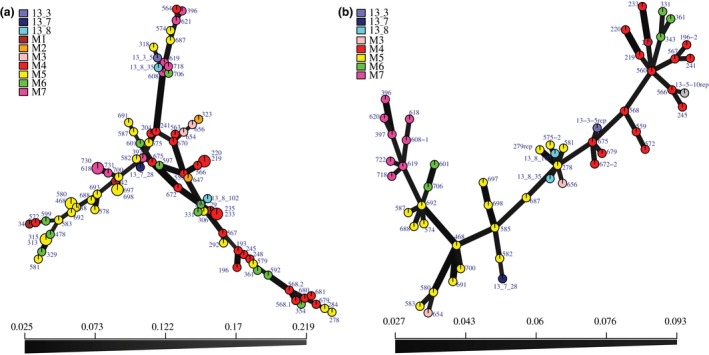
Minimum spanning networks (MSNs) inferred using SSRs (a) and SNPs (b) in 2013 and 2014 populations of *Phytophthora infestans* collected in seven fields (M1–M7) in Denmark. (a) Ten SSRs and (b) 8875 SNPs based on a Provesti's distance matrix. Each node represents a multilocus genotype (MLG), with variable size depending on the number of individuals within that MLG. The distance between the nodes represents the genetic distance between MLGs. Sampling fields are denoted by colour and  size of sample nodes represents either one or two isolates.

The unbiased index of association, *r*_d_, was significantly different (*P* < 0.001) from what is expected of a freely recombining population for all sampling fields, as well as the overall 2014 population, when calculated from the clone‐corrected SNP dataset (Table 2). However, when using the clone‐corrected SSR dataset, based on the multilocus genotypes (MLGs) defined by mlg.filter, only one sampling field had a significant *r*_d_ (*P* < 0.05), and the total population also lacked significant linkage disequilibrium (Table 2). In addition, no sign of reticulation was detected from the minimum spanning network based on a Provesti's distance matrix of the SNP dataset (Fig. 2).

**Table 2:** Unbiased index of association (*r*_d_) calculated from SSRs and SNPs datasets.

Population ID	SSRs			SNPs		
	*N*	*r*_d_	*P* value	*N*	*r*_d_	*P* value
M1	3	0.056	0.255	_	_	_
M4	17	0.012	0.431	**17**	0.014	0.001**
M5	20	0.016	0.782	**19**	0.040	0.001**
M6	11	0.084	0.059	**6**	0.034	0.001**
M7	8	0.117	0.021**	**7**	0.019	0.001**
Overall 2014	68	0.015	0.747	51	0.009	0.001**

*r*_d_ were estimated for fields with at least three individuals sampled in 2014 and on the overall 2014 population (including fields with <3 individuals). Estimates for the SNP dataset were based on a thinned dataset of 514 SNPs present in all individuals and fields. *N* is the number of individuals after clone correction based on MLGs defined by mlg.filter and ** indicates significant *P* values (*P* < 0.05, permutations = 1000).

Genotype accumulation curves showed that the ten SSR loci were sufficient to describe all the MLGs present in the population, as was the pruned 514 SNP dataset used (Supporting information, Fig. S3). The 88 (total) isolates from 2013 and 2014, genotyped with ten SSRs, resulted in 83 unique MLGs. No single identical SSR genotype was found in more than one potato field, and there were no identical SSR genotypes between 2013 and 2014. When the mlg.filter function in *poppr* was used to determine the true MLGs based on a genetic threshold determined by a gap in Bruvo's genetic distance distribution, the number of MLGs was reduced from 83 to 63 in the 2014 data. Six MLGs were found more than once within fields, but regardless none were shared between fields.

Similarly, when analysing the SNP dataset, the threshold based on Nei's genetic distance was determined at 0.03, and the 49 individuals (without replicates) were sorted into 39 MLGs. The diversity statistics based on these MLGs are listed in Table 3.

**Table 3:** Indices of multilocus diversity estimated per field and for overall 2014 population based on the contracted MLGs determined by mlg.filter for both the SSR and SNP datasets.

Population ID	SSRs					SNPs				
	*N*	MLGs	*H*	*λ*	*H*_exp_	*N*	MLGs	*H*	*λ*	*H*_exp_
M1	3	3	1.099	0.667	0.647	**_**	**_**	**_**	**_**	**_**
M2	2	2	0.693	0.500	0.500	**_**	**_**	**_**	**_**	**_**
M3	2	2	0.693	0.500	0.283	2	2	0.693	0.500	0.287
M4	25	17	2.775	0.926	0.418	16	11	2.559	0.922	0.359
M5	28	20	2.828	0.934	0.449	18	15	2.322	0.833	0.386
M6	12	11	2.369	0.903	0.472	5	4	1.332	0.720	0.329
M7	11	8	2.020	0.860	0.447	8	7	1.906	0.844	0.368
Overall 2014	83	63	4.075	0.981	0.467	49	39	3.719	0.973	0.277

*N* corresponds to number of individuals per field in the 2014 population, MLGs is the number of multilocus genotypes, *H* is the Shannon–Wiener index of diversity (Shannon, 2001), *λ* is Simpson's complement index of genotypic diversity (Simpson, 1949) and *H*_exp_ is the Nei's unbiased gene diversity (Nei, 1978).

### Ploidy

**Figure 3:**
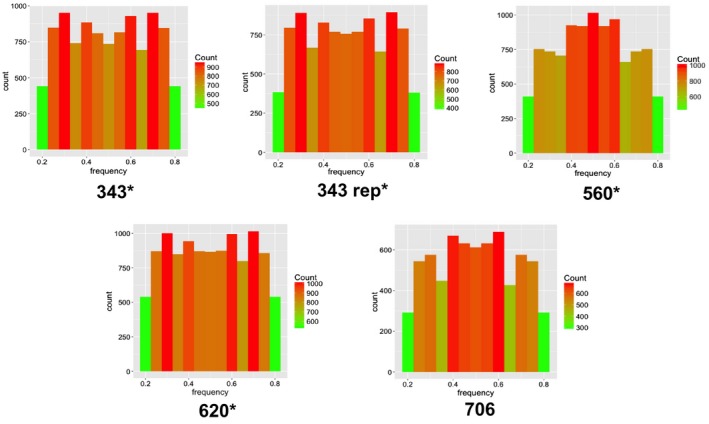
Histograms from individuals displaying a possibly non‐diploid distribution. Isolates marked with * had also one or more triploid SSR alleles.

**Figure 4:**
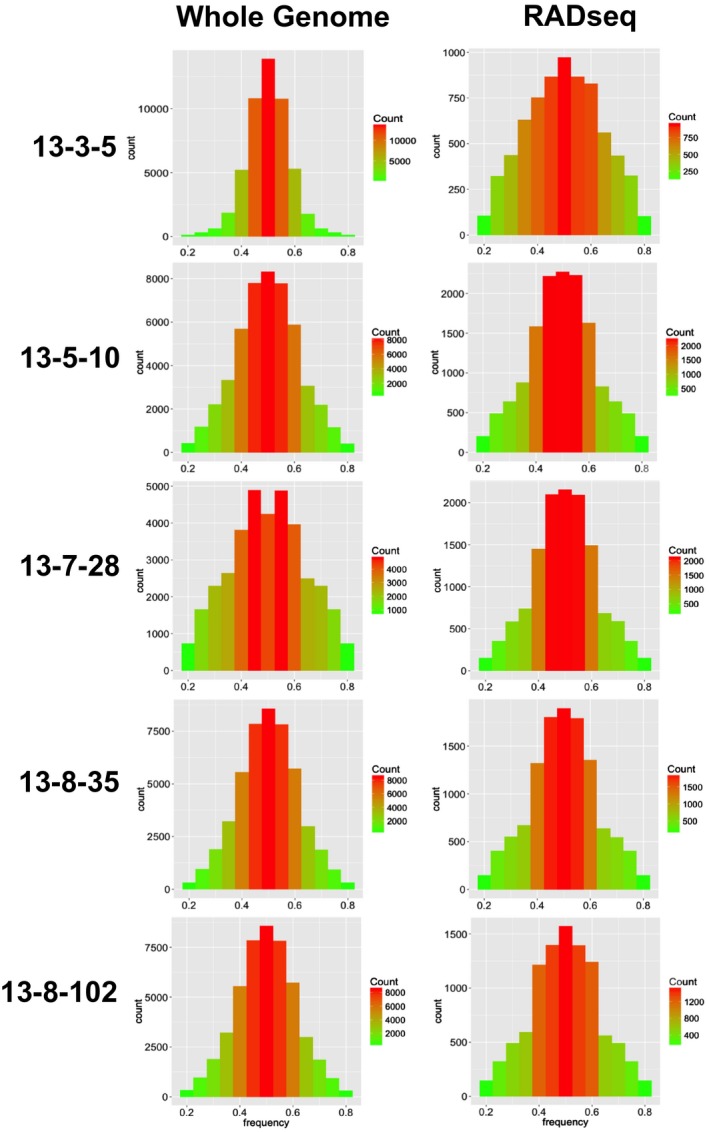
Comparison of diploid histograms generated from whole‐genome (left) and RAD (right) sequencing of five strains. Lower SNPs counts (*y*‐axes) resulting from RAD‐seq were inferred in the ploidy analysis.

Comparison of ploidy histograms, generated from whole genome and RAD sequencing, for the five isolates (13‐3‐5, 13‐5‐10, 13‐7‐28, 13‐8‐35 and 13‐8‐102) collected in Denmark in 2013 clearly showed that the sequence coverage provided by RAD‐seq ranged from approximately one‐third to one‐tenth that of the whole genome sequencing (Fig. 3). A lower sequence coverage is due to a larger number of samples multiplexed in the sequencing lanes, but yet sufficient to consistently determine ploidy in these five individuals. When we included the data from the individuals used by Yoshida *et al.* (2013) in our analysis, the test was not powerful enough to differentiate between triploids and tetraploids, but could clearly distinguish diploids from polyploids. Ploidy analysis run on all isolates included in this study revealed that the majority of the Danish samples collected in 2014 had a biallelic frequency distribution with a single peak around 0.5, as expected for diploid individuals (Fig. S4). Only four out of the 56 Danish isolates indicated a pattern of unclear ploidy or polyploidy (Fig. 4). In three out of these four isolates, one or more triploid alleles were also detected in the SSR analysis. However, in three other isolates (585, 279, 722) triploid SSR alleles were also detected, but the ploidy histograms were clearly diploid, as confirmed by the replicates. The Mexican outgroup samples also showed a diploid distribution. A histogram for each individual is included in the Supporting information (Fig. S4).

## Discussion

**Figure 5:**
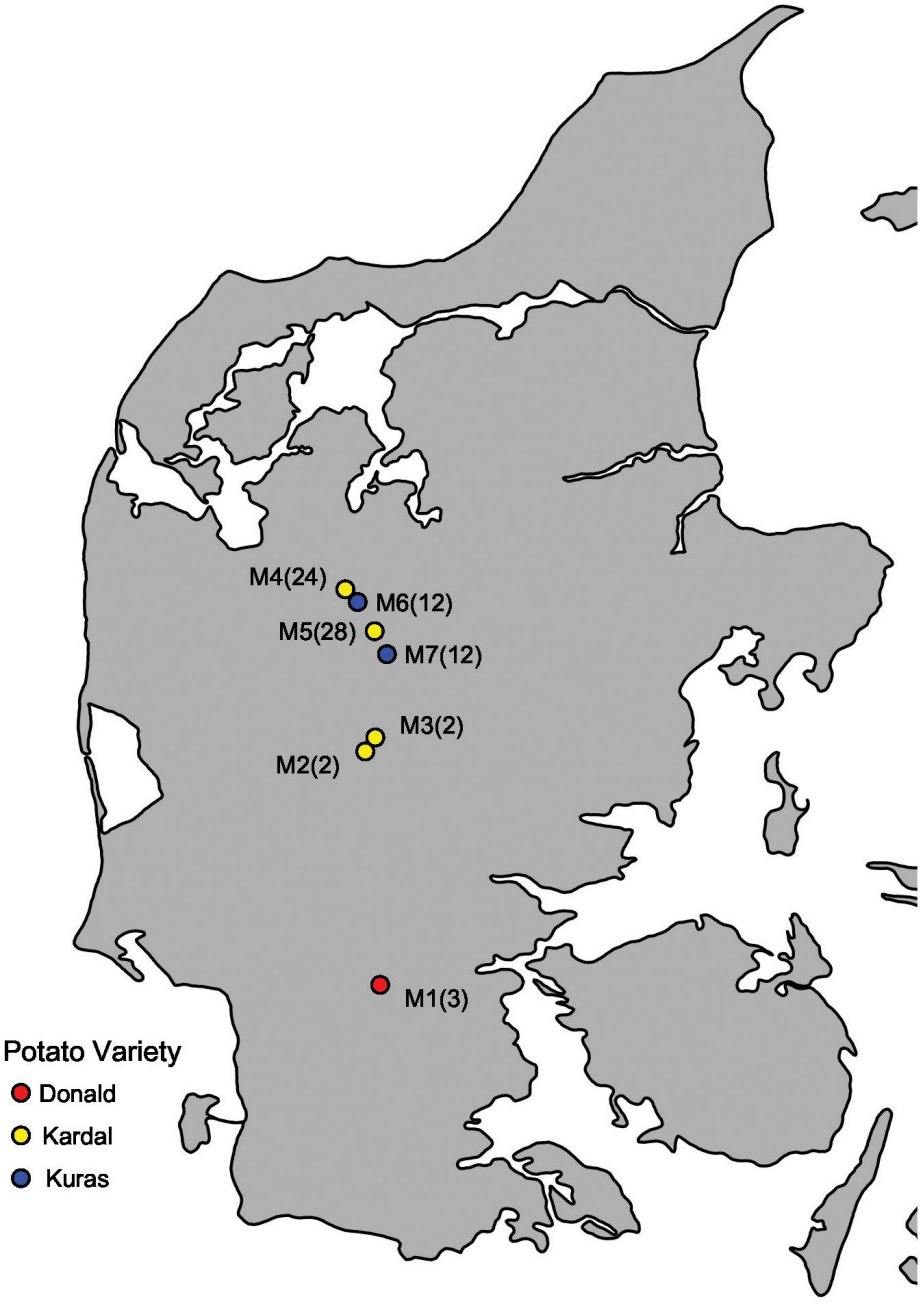
Map of field locations within Denmark. Seven fields (M1–M7) were sampled four times each throughout the 2014 growing season. The total number of samples from each field is given in parentheses and the colour denotes the potato variety.

The population structure of the 2014 outbreak of *P. infestans* in Denmark revealed a geographic structure at field level as shown by the DAPC analyses and the significant *F*_ST_ values (*P* < 0.01). None of the MLGs based on SNPs data were found in more than one field, even later in the season when asexual sporangia should have had sufficient time to spread across fields. This finding is in concordance with what was observed in the same potato‐growing region the previous year, where the measure for differentiation, *θ*, was significant between fields (Montes *et al.*, 2016). Despite the field‐level subdivision, there was no significant signal of isolation by distance (*P* = 0.114). Instead, the DAPC analyses suggest that focal outbreaks are originating from individual fields. Clear grouping by field with only rare instances of admixture was also seen in the minimum spanning network (Fig. 2). The sporangia of *P. infestans* are lightweight and have the ability to spread aerially up to 20 km in 3 h at wind speeds of 1–2 m/s (Aylor *et al.*, 2001). Though our data did not suggest that there is gene flow between neighbouring potato fields in the Danish population, the low sampling depth and large population size may leave possible gene flow undetected. The potato variety differs between the potato fields nearest one another sampled in this study (Fig. 5), but it has been shown earlier that potato cultivar *per se* is insufficient to promote local adaptation of *P. infestans*, particularly if there is gene flow between populations or if a variety is locally dominant (Montarry *et al.*, 2008). Likewise, in our region, Kuras is the dominant variety (*c*. 60–70% Kuras in 2014; KMC, Denmark, personal communication). Local adaptation to potato cultivars has only been seen over a much larger geographic distance, i.e. between France and Morocco (Andrivon *et al.*, 2007). However, it could be that unmeasured variables, such as fungicide use, account for the observed pattern of local adaptation. In general, this structure speaks against any single large uniform selection pressure in the region, or to extremely restricted gene flow (Montarry *et al.*, 2008), which may also explain why no dominant genotype is taking over in the fields sampled in Denmark, as is the case in other regions of Europe.

Interestingly, the DAPC analysis revealed a slight separation between 2013 and 2014 populations (Fig. S5), with no genotypes shared between years, and with new alleles detected in 2014. The difference between years has important implications when it comes to management, as the genetic make‐up of one year's epidemic may not be used to predict the nature of the outbreak in the coming year. However, the exact same fields were not tested in 2013 and 2014, due to crop rotation practices, and alleles can be missed due to chance alone, taking the low sampling intensity into consideration.

All but four of the 56 Danish individuals characterized in this study displayed a strong signal of diploidy (Figs 3 and 4). Yoshida *et al.* (2013) were the pioneers in using the allelic ratios of biallelic SNPs to determine ploidy in whole genomes of *P. infestans*. They found that the majority of modern‐day samples were triploid, while historic herbarium samples were generally diploid. Older techniques that quantified DNA content based on cytological, cytometric and/or fluorescence techniques found that Mexican samples were consistently diploid, but European samples showed a great variability in ploidy, with the majority of modern samples being polyploid or diploid heterokaryons (Ritch and Daggett, 1995; Therrien *et al.*, 1989; Tooley and Therrien, 1987; Whittaker *et al.*, 1991). The presence of some triploid SSR alleles in a small number of both polyploid and diploid individuals in this study suggests that there may be some aneuploidy present in the Danish population as well, and that it may not have been detected by the SNP‐based technique or  stacks pipeline used in this study. They could, however, also represent PCR errors or smaller regions of the genome that display copy number variation, rather than whole chromosome duplication. Although the samples shown in Fig. 3 are clearly not strict diploids, one could argue that they are not clearly triploid either, and may be more indicative of heterokaryosis.

The presence of diploid individuals in Denmark implies that the population maintains an ability for sexual reproduction, compared to triploids in which the uneven number of chromosomes may act as a barrier to sex. Li *et al.* (2017) recently found that *P. infestans* populations were primarily triploid in clonal populations and diploid in populations presumed to be sexually reproducing based on high levels of genotypic diversity. They also found that individuals had the ability to switch from triploid to diploid when exposed to stress, such as a non‐lethal dose of the fungicide metalaxyl (Li *et al.*, 2017). This suggests that the genome of *P. infestans* has some flexibility that could pose additional challenges for its control. It has been suggested that cold winters, such as in Scandinavia, may favour sexual reproduction due to the more durable structures of the sexually produced oospores which can survive in the soil (Sjöholm *et al.*, 2013); however, there have been many instances reported of self‐fertile *P. infestans* individuals that do not require sex to produce oospores (Campbell *et al.*, 1985; Han *et al.*, 2013; Pipe *et al.*, 2000; Shattock *et al.*, 1986; Smart *et al.*, 1998; Tantius *et al.*, 1986). The findings of Li *et al.* (2017) that diploidy can be induced using metalaxyl suggest that the maintenance of sex may not have to do with the durable resting structures produced after all, but rather the increased ability to adapt to all nature of stresses.

Because the presence of oospores alone does not necessarily guarantee that sexual reproduction is occurring or successfully contributing to epidemics, it is important to test for signals of sexual recombination when describing the population structure of *P. infestans*. The unbiased index of association, *r*_d_, was significantly different from what is expected of a freely recombining population for all fields when calculated from the SNP dataset (Table 2). Past studies of Scandinavian populations have shown mixed results in regard to the index of association, with some showing an overall significant *I*_A_ (*P* < 0.001; Brurberg *et al.*, 2011) and others a lack of significance for the overall population (*P* = 0.73 in Denmark), but significant *I*_A_ at the field level for only some fields (Sjöholm *et al.*, 2013). In a 2013 analysis of the Danish population, the *I*_A_ was also significant (*P* < 0.001), suggesting that the population is clonal (Montes *et al.*, 2016). However, these previous studies are all based on SSRs. The SSR data in the current study only produced a significant *r*_d_ value at one field, M7 (Table 2). Although a single SSR locus carries more information than a single SNP, there are many unknowns still surrounding SSR loci, e.g. how they are spread throughout the genome, how to properly describe their mutation model with regard to the possibility for homoplasy, and the biases in mutation rate that could exist depending on the heterozygosity, size and location of the SSR locus (Amos, 2010, 2016; Defaveri *et al.*, 2013; Jarne and Lagoda, 1996). SSRs have also received criticism for their high rate of genotyping error (Defaveri *et al.*, 2013). In comparison, SNPs have a straightforward mutation model, and as they are high in number and are dispersed randomly throughout the entire genome, have an advantage over the limited number of SSR loci (Defaveri *et al.*, 2013).

The *F*_IS_, which was negative for all populations, also points towards clonal reproduction (Table 1). *F*_IS_ is a sensitive measure of clonality in acyclic parasite populations, as even a low level of sexual reproduction in a population is expected to quickly bring the *F*_IS_ back towards zero (Prugnolle and De Meeûs, 2008). Significant excesses of heterozygosity, as seen here (Fig. S1), are only expected for populations with extreme rates of clonality (Balloux *et al.*, 2003) due to the accumulation of mutations through successive rounds of asexual reproduction. Li *et al.* (2017) found that in samples that changed from triploid to diploid under stress conditions, heterozygosity was maintained at the higher triploid level in the newly formed diploids. However, if the switch to diploidy accompanied a switch to sexual reproduction, recombination would act as homogenizing force over time, decreasing heterozygosity and bringing the *F*_IS_ rapidly back to zero (Balloux *et al.*, 2003).

The minimum spanning network shows a structure with no reticulation, as one would expect from a pathogen that is spreading clonally (Fig. 2). Not only is there a separation between fields, but one also sees how the outbreak changes over time. The MLG at the centre of the network consists of isolates 278 and 279 from field M5, isolated from the first collection date. At the next sampling date of the same plots, these are replaced by closely related genotypes that have most likely arisen through mutation during asexual reproduction of the original MLGs, as several generations of asexual reproduction are expected to occur within a growing season.

The genotypic diversity found in the current study (Table 1) was comparable to what has been found for individual Scandinavian countries in the past (Brurberg *et al.*, 2011). This genotypic diversity is high when compared to countries like Ireland, where a few aggressive clonal lineages usually dominate each year (Cooke *et al.*, 2012; Li *et al.*, 2013b; Sjöholm *et al.*, 2013). Genotypic diversity is the only measure of sexuality that increases constantly with the amount of sexual reproduction in partially clonal populations (Balloux *et al.*, 2003). However, the presence of highly diverse populations in areas where only one *P. infestans* mating type exists, and sexual reproduction should theoretically be impossible (Barquero *et al.*, 2005; Delgado *et al*., 2013; Li *et al*., 2013b), begs the question whether another mechanism for generating such diversity might exist. It has been suggested, for example, that mitotic gene conversion could play a role in generating the high diversity seen in asexually produced *P. infestans* progeny (Abu‐El Samen *et al.*, 2003).

Tajima's *D* was positive for all populations tested (Table 1). Assuming a constant effective population size, this means that balancing selection is acting on the genome, as opposed to directional selection imposed by a single selective pressure such as a fungicide or a single plant resistance (*R*) gene. Tajima's *D* is sensitive to demographic changes in populations, however, so that an alternative explanation for a positive Tajima's *D* is a contraction in population size, such as a recent bottleneck. If the population is primarily overwintering clonally in tubers, then one would indeed expect to see an annual bottleneck between growing seasons, as only certain strains would survive. A trade‐off has been observed in *P. infestans* between virulence and the ability to overwinter in tubers, as overly aggressive strains that may be the most successful during the summer growing season will kill their host tubers and not survive the winter (Pasco *et al.*, 2016). The extent to which these different factors contribute to the positive Tajima's *D* may therefore warrant further study.

## Experimental Procedures

### Sample collection

Seven potato fields in Denmark were visited at the earliest signs of infection during summer 2014, and again three more times, about a month apart, throughout the growing season for a total of four collection dates per field (Fig. 5). Within each field, four different outbreak epicentres were sampled, and four potato plants within each of these epicentres where it was possible. In addition, during the first sampling, individual plants were sampled four times from lesions on different parts of the plant. At the time of the last sampling field 1 was harvested, and only a single outbreak was seen in field 4. Out of 621 collections, a total of 83 strains of *P. infestans* was successfully isolated, maintained and subjected to further testing. Isolates' ID, sampling date and localities are listed in Table S1. Additionally, five isolates from 2013 and two Mexican isolates were included in the analyses.

Isolation was performed by plating leaves with a lesion on rye agar. Once a lesion started sporulating, single sporangia were transferred to new Petri dishes and subcultured several times over the coming weeks to ensure that there was only one isolate per sample. Purified cultures were then stored on rye or pea agar at 4 °C until used.

### SSR analysis

DNA was extracted from axenic cultures of *P. infestans* and extracted with the QuickExtract™ Plant DNA extraction kit (Epicentre). Ten of the 12 SSR loci described by Y. Li *et al.* (2013a), were used in the analysis (see Supplementary information Text S1 on details of the analysis). MLGs identified using GeneMapper v 5.0 (Applied Biosystems, 2005) are listed in Table S2. In addition to the 83 new samples collected in 2014, microsatellite data from 84 samples collected in 2013 and previously analysed by Montes *et al.* (2016) were included in some analyses (see the Population genetic analyses section).

### Whole genome sequencing

Six individuals collected from Denmark in 2013, and genotyped with ten SSR markers in a previous study (Montes *et al.*, 2016), were whole‐genome sequenced (Table S1). In order to ensure at least one sample with a high coverage, sample 13‐3‐5 was chosen to make up the largest portion (*c*. 33.3%) of the final pool, whereas the other five samples each made up approximately 13.3% (see Supplementary information Text S1 on details of library preparation). Libraries were sequenced at the Danish National High‐Throughput DNA Sequencing Centre on an Illumina HiSeq 2000 platform using 150 bp paired end reads (see Supplementary information Text S1 for details).

### Restriction‐site Associated DNA sequencing, RAD‐seq

A total of 78 samples were included in the RAD‐seq study, including 58 individuals collected in Denmark in 2014, the five individuals isolated in 2013 for which whole‐genome sequencing was also performed, two individuals from Mexico to serve as an outgroup, nine biological replicates and four technical replicates (Table S1). The single‐digest RAD libraries were prepared according to the original protocol of Baird *et al.* (2008), with some modifications for fungal DNA (see Supplementary information Text S1 for details on preparation of RAD libraries). The six prepared libraries were amplified in 25 µL reaction volumes, and the PCR products were cleaned with AMPure beads. Libraries were quantified using qPCR, pooled at equimolar concentrations into groups of 39 samples and paired‐end sequenced using two lanes of an Illumina HiSeq 2500 at the Norwegian Sequencing Centre. Both biological and technical replicates were prepared and sequenced in separate libraries. Six samples were not successfully sequenced (313, 315, 592, 597, 723 and 730), likely due to a problem with the P1 adapter as they all had the same one.

### Preparation of RAD‐seq data

The *P. infestans* T30‐4 reference genome (Haas *et al.*, 2009) was corrected using the 13‐3‐5 whole genome sequence from Denmark in 2013 in order to create a reference that more closely represents the Danish population as follows. The reads from the 13‐3‐5 Danish isolate were first processed for library adapter removal and initial filtering using Trimmomatic v. 0.32. The MAXINFO parameter was used to prioritize keeping reads at a length of 75 bp or higher at the expense of quality (ILLUMINACLIP:adapters.fa:2:30:10 LEADING:3 TRAILING:3 MAXINFO:75:0.8 MINLEN:70) as suggested for downstream analysis using Pilon v. 1.17 (Walker *et al.*, 2014). The remaining paired and unpaired reads were aligned to the T30‐4 reference genome using Bowtie2 v. 2.1.0 (Langmead and Salzberg, 2012). The alignment files were sorted and indexed using SAMtools v. 1.3 (Li *et al*., 2009). Pilon v. 1.17 was used to change single bases in the reference genome to more closely represent the Danish population (Walker *et al.*, 2014). Insertion‐deletions or gaps larger than a single base were not modified due to memory limitations. The resulting ‘fixed’ reference was used to align the RAD‐seq reads.

RAD‐seq data was analysed using the pipeline stacks v. 1.39 (Catchen *et al.*, 2013). First, the *clone_filter* program was run to identify and discard PCR clones, which eliminated 34.88% of the reads (157 635 363 pairs of reads). The filtered reads were de‐multiplexed using *process_radtags*. Out of 588 582 208 sequences input, 100 963 218 were dropped due to ambiguous barcodes, 166 923 due to low quality (MQ < 10, based on a sliding window 0.15 of the read length), and 5 258 815 due to ambiguous RAD‐Tags, resulting in a total of 482 193 252 retained reads (82% of the filtered data). Four individuals (isolates 323, 647, 599 and 13‐3‐5) were removed due to low sequence coverage (under 10×), therefore 68 samples were retained overall.

Alignments were performed against the corrected reference genome using Bowtie2, with parameters specifying that each read should be aligned to only one location in the genome, and not allowing for discordant mapping, the mapping of a single read without its mate pair or unaligned reads. The aligned reads were passed to the refmap.pl programme in stacks and filtered in the populations module using optimized key parameters based on mean coverage and number of resulting SNPs as follows: a minimum stack depth of 10, SNPs found in at least 50% of the individuals in a population for that population to be included and SNPs present in seven out of the ten populations to be included. This resulted in a dataset of 55 288 SNPs with a mean coverage *c*. 30× across individuals, henceforth referred to as the ‘full dataset’. To account for physical linkage, the full dataset was further filtered using vcftools (Danecek *et al*., 2011) and resulted in 8875 SNPs separated by a minimum of 1000 bp (thinned), and a third pruned dataset with no missing data, consisting of 514 SNPs present in all individuals of all populations, was used to estimate the index of association (see section  population genetic analyses).

### Population genetic analyses

Both the SSR and the RAD‐seq‐generated SNP datasets were analysed using the package poppr v. 2.3.0 (Kamvar *et al.*, 2014, 2015) in R v. 3.2.3 (R Core Team, 2015). For the SSR analyses, all loci were treated as triploid in order to include a third allele in those individuals and loci where one was present, but treating the third allele as a null allele in the individuals where one was not. A genotype accumulation curve was first plotted to ensure that the number of loci suitably captured all the variation present in the populations. The *mlg.filter* function was activated to determine the true number of MLGs by using a genetic distance threshold determined using the *cutoff_predictor* tool, which finds a gap in the distance distribution. For SSRs we estimated the Bruvo's distance (Bruvo *et al.*, 2004), while for SNPs we used Nei's distance (Nei, 1978). For the 12 replicates (biological and technical) included in the study, once we checked that the pairs clustered together in all analyses (see Fig. S2), we re‐ran the analyses keeping only the higher‐coverage replicate. Analyses carried out in *poppr*, on the SSR and SNP datasets, included a summary of diversity measures, a Mantel test for isolation by distance, genetic distance matrices, and minimum spanning trees and networks based on distance matrices.

*F*‐statistics were calculated using the hierfstat v. 0.04‐22 package (Goudet and Jombart, 2015) in R. Only individuals with a maximum of 25% of missing data were included. Overall *F*_ST_, *F*_IS_ and *H*_t_ (gene diversity) were calculated for the 2014 population, as well as pairwise *F*_ST_ between fields based on Nei's estimator. The significance of the *F*_ST_ structure between populations was tested using the *G*‐statistic test (1000 permutations) including only SNPs with a maximum of 2% of missing loci. The inbreeding coefficient *F*_IS_ and mean gene diversity *H*_S_ were also estimated within each sampling field. Tajima's *D* was calculated in a 50 kb window using PopGenome (Pfeifer *et al.*, 2014).

The index of association (*I*_A_) was calculated in poppr to test for linkage disequilibrium based on a clone‐corrected dataset. A modification of *I*_A_ that removes the bias of sample size, *r*_d_ (Agapow and Burt, 2001), was also calculated. For the SNP marker set, the *I*_A_ was run on the full dataset, as well as on a filtered dataset of 8875 SNPs and on a third similarly filtered dataset with only the 514 SNPs that were present in all individuals of all populations. The SNP datasets used did not change the significance of the results.

DAPC was run using the adegenet package (Jombart, 2008; Jombart and Ahmed, 2011) in R on both SSR and SNP datasets from 2013 and 2014. For the SNP dataset, in the case of individuals with replicates, only the replicate with the higher coverage was used to avoid a false pattern of clustering. The optimum number of principle components (PCs) retained for each analysis was first determined using the xvalDAPC function and 1000 replicates. After running the DAPC, posterior probabilities were used to calculate the proportion of successful reassignment of individuals to their populations and to visualize the composition of these population clusters.

### Ploidy analysis

Both datasets resulting from the whole genome and the RAD sequencing were analysed for ploidy level, based on the method developed by Yoshida *et al.* (2013). Three samples from their study with varying ploidy (M‐0182896, DDR7602, 06_3928A) were included as a control in our analysis to verify whether our SNPs calling method yielded consistent results. The Genome Analysis ToolKit (GATK v. 3.6) was used to call SNPs from only the gene‐dense regions of the T30‐4 reference genome (Haas *et al.*, 2009). Only biallelic SNPs with the following criteria were used: a mapping quality (MQ) ≥ 30, a minimum depth of coverage of 10 and allelic frequencies between 0.20 and 0.80. Allelic frequencies at each SNP were then graphed to a histogram using the ggplot2 package (Wickham, 2009) in R and compared to the expected pattern of allele frequencies as simulated by Yoshida *et al.* (2013).

## Supporting information


**Fig. S1** Observed (*H*_obs_) vs. expected (*H*_exp_) heterozygosities for each SNP locus averaged over all individuals. The *x*‐axis is the *H*_obs_ ranging from 0 to 1.0 and the *y*‐axis is the *H*_exp_ ranging from 0 to 0.5.

**Fig. S2** A UPGMA phylogenetic tree of the RAD‐seq individuals based on a Provesti's distance matrix and bootstrapped 100 times. Nodes without a bootstrap value have no bootstrap support (bootstrap =  0). Nodes are colour‐coded by field of origin.

**Fig. S3** Genotype accumulation curves showing that the number of loci used are sufficient to capture all multilocus genotypes. Top, SSR loci. Bottom, the smallest SNP dataset used, consisting of 514 SNPs present in all populations and separated by 1000 bp (used in *I*_A_ analysis).

**Fig. S4** Ploidy histograms for all isolates generated from whole genome sequencing (WGS) and from RAD seq. The last three histograms are of isolates also analysed by Yoshida *et al*. (2013), using sequence data downloaded from the EBI database. The scale of the *y*‐axis (number of reads) results from the sequence coverage and is variable for each isolate.

**Fig. S5** DAPC of 2013 and 2014 individuals based on SSR data. Top, DAPC grouped by field (labelled “year_field no.”). The first 21 PCs were retained. The 2014 samples are represented by squares and diamonds in shades of blue, while the 2013 isolates are represented by circles in shades of red. Bottom, DAPC of samples grouped by year, 2013 (red) versus 2014 (blue). The first 25 PCs were retained. As only two groups were being compared, there was a single discriminant function, plotted along the *x*‐axis.

**Table S1** List of isolates included in this study. Isolate name, sampling locality and date, and the markers generated. The five isolates that were whole‐genome sequenced are in bold. More information on these isolates can be found in Montes *et al*. (2016), where they are characterized using ten SSR markers.

**Table S2** List of SSR MLGs for each isolate from the 2014 Danish population.

**Table S3** Pairwise *F*_ST_ values between sampling fields in 2014 based on both SSR and SNP datasets.

**Text S1** SSR analysis, whole genome and RAD‐seq library building.
