# Supplementary material for: Population genomics of an outbreak of the potato late blight pathogen, Phytophthora infestans, reveals both clonality and high genotypic diversity
Source: Mol Plant Pathol. 2019 May 30;20(8):1134–46. doi: 10.1111/mpp.12819 (PMC6640178; doi:10.1111/mpp.12819)

**Figure S1.** Observed (Hobs) vs. expected (Hexp) heterozygosities for each SNP locus averaged over all individuals. With x-axis indicating Hobs that ranges from 0-1.0, while y-axis indicating Hexp ranges from 0-0.5.


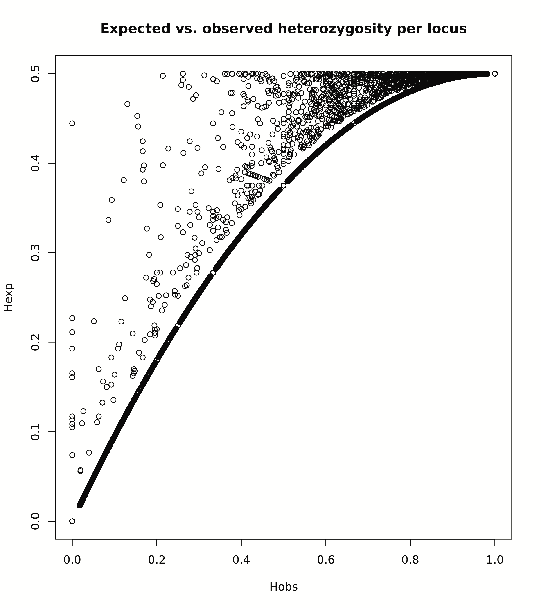

Supplement: Supplementary file 1 — Fig. S1 Observed (H obs) vs. expected (H exp) heterozygosities for each SNP locus averaged over all individuals. The x‐axis is the H obs ranging from 0 to 1.0 and the y‐axis is the H exp ranging from 0 to 0.5. [file MPP-20-1134-s001.docx]
