# Supplementary material for: Population genomics of an outbreak of the potato late blight pathogen, Phytophthora infestans, reveals both clonality and high genotypic diversity
Source: Mol Plant Pathol. 2019 May 30;20(8):1134–46. doi: 10.1111/mpp.12819 (PMC6640178; doi:10.1111/mpp.12819)

**Figure S2.** A UPGMA phylogenetic tree of the RAD-seq individuals based on a Provesti's distance matrix and bootstrapped 100 times. Nodes without a bootstrap value have no bootstrap support (bootstrap=0). Nodes are color-coded by field of origin.


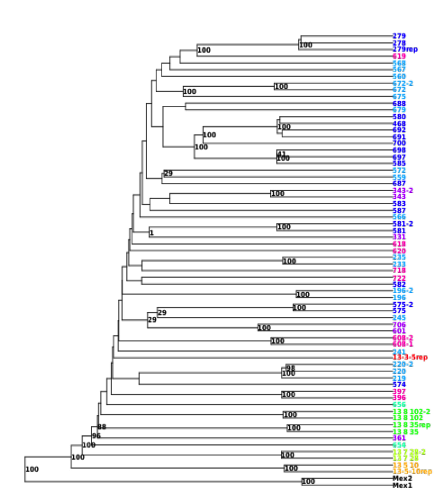

Supplement: Supplementary file 2 — Fig. S2 A UPGMA phylogenetic tree of the RAD‐seq individuals based on a Provesti's distance matrix and bootstrapped 100 times. Nodes without a bootstrap value have no bootstrap support (bootstrap = 0). Nodes are colour‐coded by field of origin. [file MPP-20-1134-s002.docx]
