# Supplementary material for: Population genomics of an outbreak of the potato late blight pathogen, Phytophthora infestans, reveals both clonality and high genotypic diversity
Source: Mol Plant Pathol. 2019 May 30;20(8):1134–46. doi: 10.1111/mpp.12819 (PMC6640178; doi:10.1111/mpp.12819)

**Figure S3.** Genotype accumulation curves showing that the number of loci used are sufficient to capture all multi-locus genotypes. (Top) SSR loci. (Bottom) The smallest SNP dataset used, consisting of 514 SNPs present in all populations and separated by 1000 bp (used in I_A_ analysis).


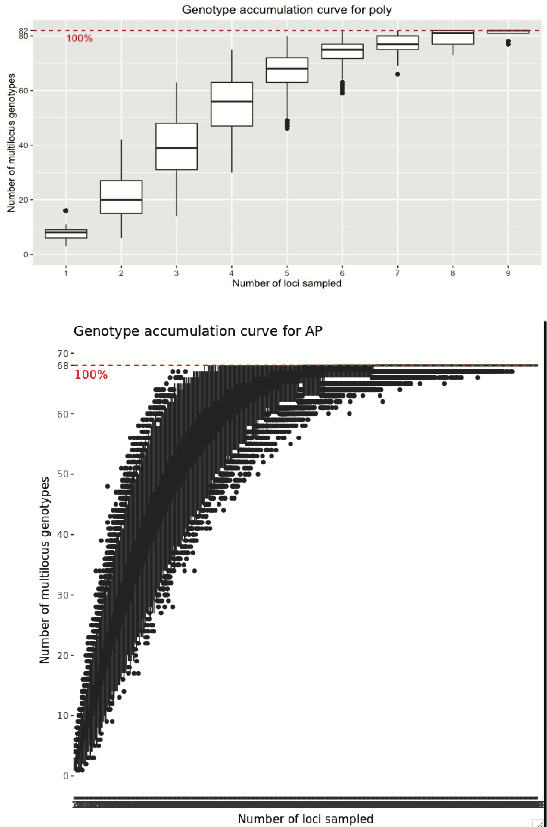

Supplement: Supplementary file 3 — Fig. S3 Genotype accumulation curves showing that the number of loci used are sufficient to capture all multilocus genotypes. Top, SSR loci. Bottom, the smallest SNP dataset used, consisting of 514 SNPs present in all populations and separated by 1000 bp (used in I A analysis). [file MPP-20-1134-s003.docx]
