# Supplementary material for: Population genomics of an outbreak of the potato late blight pathogen, Phytophthora infestans, reveals both clonality and high genotypic diversity
Source: Mol Plant Pathol. 2019 May 30;20(8):1134–46. doi: 10.1111/mpp.12819 (PMC6640178; doi:10.1111/mpp.12819)
Supplement: Supplementary file 4 — Fig. S4 Ploidy histograms for all isolates generated from whole genome sequencing (WGS) and from RAD seq. The last three histograms are of isolates also analysed by Yoshida et al. (2013), using sequence data downloaded from the EBI database. The scale of the y‐axis (number of reads) results from the sequence coverage and is variable for each isolate. [file MPP-20-1134-s004.docx]

**Figure S4.** Ploidy histograms for all isolates generated from whole-genome sequencing (WGS) and from RAD-seq. The last three histograms are of isolates also analyzed by Yoshida *et al*. (2013), using sequence data downloaded from the EBI database. The scale of the y-axis (number of reads) result from the sequence coverage and is variable for each isolate.
