# Supplementary material for: Population genomics of an outbreak of the potato late blight pathogen, Phytophthora infestans, reveals both clonality and high genotypic diversity
Source: Mol Plant Pathol. 2019 May 30;20(8):1134–46. doi: 10.1111/mpp.12819 (PMC6640178; doi:10.1111/mpp.12819)

**Figure S5**. DAPC of 2013 and 2014 individuals based on SSR data. **Top:** DAPC grouped by field (labelled “year_field-no.”). The first 21 PCs were retained. The 2014 samples are represented by squares and diamonds in shades of blue, while the 2013 isolates are represented by circles in shades of red. **Bottom:** DAPC of samples grouped by year, 2013 (red) vs 2014 (blue). The first 25 PCs were retained. As only two groups were being compared, there was a single discriminant function, plotted along the x-axis.


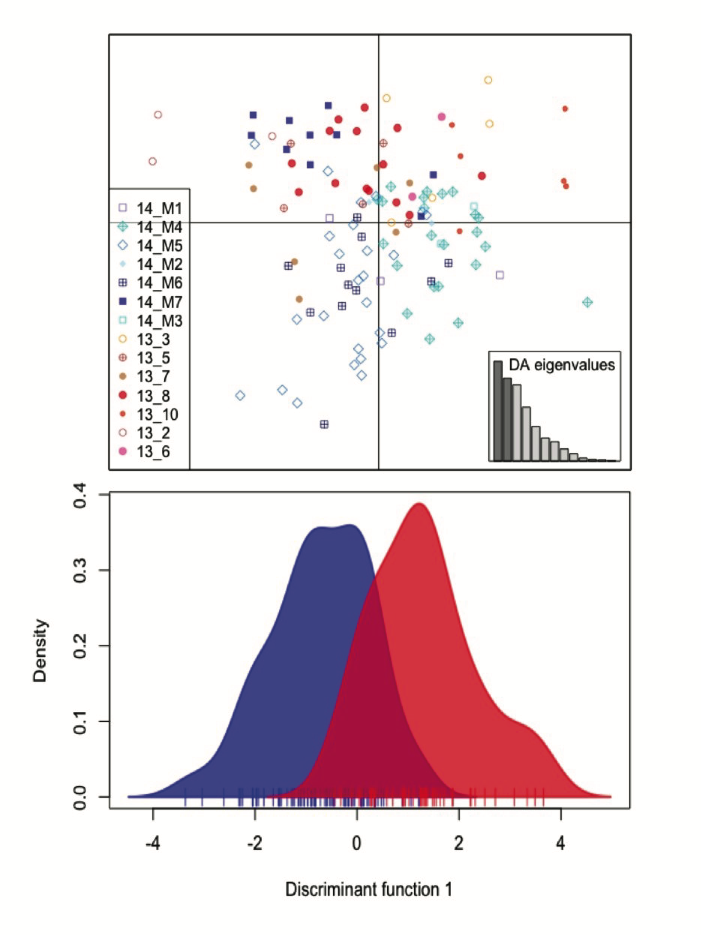

Supplement: Supplementary file 5 — Fig. S5 DAPC of 2013 and 2014 individuals based on SSR data. Top, DAPC grouped by field (labelled “year_field no.”). The first 21 PCs were retained. The 2014 samples are represented by squares and diamonds in shades of blue, while the 2013 isolates are represented by circles in shades of red. Bottom, DAPC of samples grouped by year, 2013 (red) versus 2014 (blue). The first 25 PCs were retained. As only two groups were being compared, there was a single discriminant function, plotted along the x‐axis. [file MPP-20-1134-s005.docx]
