# Supplementary material for: Population genomics of an outbreak of the potato late blight pathogen, Phytophthora infestans, reveals both clonality and high genotypic diversity
Source: Mol Plant Pathol. 2019 May 30;20(8):1134–46. doi: 10.1111/mpp.12819 (PMC6640178; doi:10.1111/mpp.12819)
Supplement: Supplementary file 6 — Table S1 List of isolates included in this study. Isolate name, sampling locality and date, and the markers generated. The five isolates that were whole‐genome sequenced are in bold. More information on these isolates can be found in Montes et al. (2016), where they are characterized using ten SSR markers. [file MPP-20-1134-s006.docx]

**Table S1.** List of isolates included in this study. Isolate name, sampling locality and date and the markers generated. The five isolates that were whole-genome sequenced were bolded. More information on these isolates can be found in (Montes *et al*., 2016), where they were characterized using 10 SSR markers.

| **Isolate ID** | **Field** | **Sample data** | **Marker set applied** |
| --- | --- | --- | --- |
| 29 | M1 | JUNE 2014 | SSR |
| 34 | M1 | JUNE 2014 | SSR |
| 42 | M1 | JUNE 2014 | SSR |
| 193 | M4 | JUNE 2014 | SSR |
| 196^B^ | M4 | JUNE 2014 | SSR+RAD |
| 204 | M4 | JUNE 2014 | SSR+RAD |
| 219 | M4 | JUNE 2014 | SSR+RAD |
| 220^B^ | M4 | JUNE 2014 | SSR+RAD |
| 233 | M4 | JUNE 2014 | SSR+RAD |
| 235 | M4 | JUNE 2014 | SSR+RAD |
| 241 | M4 | JUNE 2014 | SSR+RAD |
| 245 | M4 | JUNE 2014 | SSR+RAD |
| 248 | M4 | JUNE 2014 | SSR |
| 278 | M5 | JUNE 2014 | SSR+RAD |
| 279^C^ | M5 | JUNE 2014 | SSR+RAD |
| 284 | M5 | JUNE 2014 | SSR |
| 292 | M5 | JUNE 2014 | SSR |
| 306 | M5 | JUNE 2014 | SSR |
| 313 | M5 | JUNE 2014 | SSR+RAD |
| 315 | M5 | JUNE 2014 | SSR+RAD |
| 318 | M5 | JUNE 2014 | SSR |
| 323 | M2 | JUNE 2014 | SSR+RAD |
| 329 | M6 | JUNE 2014 | SSR |
| 331 | M6 | JUNE 2014 | SSR+RAD |
| 343^B^ | M6 | JUNE 2014 | SSR+RAD |
| 354 | M6 | JUNE 2014 | SSR |
| 361 | M6 | JUNE 2014 | SSR+RAD |
| 396 | M7 | JUNE 2014 | SSR+RAD |
| 397 | M7 | JUNE 2014 | SSR+RAD |
| 468 | M5 | JULY 2014 | SSR+RAD |
| 478 | M6 | JULY 2014 | SSR |
| 559 | M4 | AUGUST 2014 | SSR+RAD |
| 560 | M4 | AUGUST 2014 | SSR+RAD |
| 563 | M4 | AUGUST 2014 | SSR |
| 564 | M4 | AUGUST 2014 | SSR |
| 566 | M4 | AUGUST 2014 | SSR+RAD |
| 567 | M4 | AUGUST 2014 | SSR+RAD |
| 568 | M4 | AUGUST 2014 | SSR+RAD |
| 572 | M4 | AUGUST 2014 | SSR+RAD |
| 574 | M5 | AUGUST 2014 | SSR+RAD |
| 575^B^ | M5 | AUGUST 2014 | SSR+RAD |
| 578 | M5 | AUGUST 2014 | SSR |
| 579 | M5 | AUGUST 2014 | SSR |
| 580 | M5 | AUGUST 2014 | SSR+RAD |
| 581^B^ | M5 | AUGUST 2014 | SSR+RAD |
| 582 | M5 | AUGUST 2014 | SSR+RAD |
| 583 | M5 | AUGUST 2014 | SSR+RAD |
| 585 | M5 | AUGUST 2014 | SSR+RAD |
| 587 | M5 | AUGUST 2014 | SSR+RAD |
| 588 | M5 | AUGUST 2014 | SSR+RAD |
| 592 | M6 | AUGUST 2014 | SSR+RAD |
| 594 | M6 | AUGUST 2014 | SSR |
| 597 | M6 | AUGUST 2014 | SSR+RAD |
| 599 | M6 | AUGUST 2014 | SSR+RAD |
| 601 | M6 | AUGUST 2014 | SSR+RAD |
| 608^B^ | M7 | AUGUST 2014 | SSR+RAD |
| 618 | M7 | AUGUST 2014 | SSR+RAD |
| 619 | M7 | AUGUST 2014 | SSR+RAD |
| 620 | M7 | AUGUST 2014 | SSR+RAD |
| 621 | M7 | AUGUST 2014 | SSR |
| 647 | M2 | SEPTEMBER 2014 | SSR+RAD |
| 654 | M3 | SEPTEMBER 2014 | SSR+RAD |
| 656 | M3 | SEPTEMBER 2014 | SSR+RAD |
| 670 | M4 | SEPTEMBER 2014 | SSR |
| 672^B^ | M4 | SEPTEMBER 2014 | SSR+RAD |
| 675 | M4 | SEPTEMBER 2014 | SSR+RAD |
| 679 | M4 | SEPTEMBER 2014 | SSR+RAD |
| 680 | M4 | SEPTEMBER 2014 | SSR |
| 681 | M4 | SEPTEMBER 2014 | SSR |
| 687 | M5 | SEPTEMBER 2014 | SSR+RAD |
| 688 | M5 | SEPTEMBER 2014 | SSR+RAD |
| 691 | M5 | SEPTEMBER 2014 | SSR+RAD |
| 692 | M5 | SEPTEMBER 2014 | SSR+RAD |
| 693 | M5 | SEPTEMBER 2014 | SSR |
| 697 | M5 | SEPTEMBER 2014 | SSR+RAD |
| 698 | M5 | SEPTEMBER 2014 | SSR+RAD |
| 700 | M5 | SEPTEMBER 2014 | SSR+RAD |
| 706 | M6 | SEPTEMBER 2014 | SSR+RAD |
| 718 | M7 | SEPTEMBER 2014 | SSR+RAD |
| 722 | M7 | SEPTEMBER 2014 | SSR+RAD |
| 723 | M7 | SEPTEMBER 2014 | RAD |
| 730 | M7 | SEPTEMBER 2014 | SSR+RAD |
| 731 | M7 | SEPTEMBER 2014 | SSR |
| Mex1 | Mexico |  | RAD |
| Mex2 | Mexico |  | RAD |
| 13_3_5^AC^ | M13 | JUNE 2013 | SSR+RAD+WGS |
| 13_5_10^AC^ | M15 | JULY 2013 | SSR+RAD+WGS |
| 13_7_28^AB^ | M17 | JULY 2013 | SSR+RAD+WGS |
| 13_7_33^AC^ | M18 | JULY 2013 | SSR+WGS |
| 13_8_35^AC^ | M18 | JULY 2013 | SSR+RAD+WGS |
| 13_8_102^AB^ | M18 | SEPTEMBER 2013 | SSR+RAD+WGS |
| ^A^Details and SSR results in Montés et al., 2016 |  |  |  |
| ^B^Biological replicates |  |  |  |
| ^C^ Technical replicates |  |  |  |
