# Supplementary material for: Population genomics of an outbreak of the potato late blight pathogen, Phytophthora infestans, reveals both clonality and high genotypic diversity
Source: Mol Plant Pathol. 2019 May 30;20(8):1134–46. doi: 10.1111/mpp.12819 (PMC6640178; doi:10.1111/mpp.12819)
Supplement: Supplementary file 7 — Table S2 List of SSR MLGs for each isolate from the 2014 Danish population. [file MPP-20-1134-s007.docx]

**Table S2.** List of SSR MLGs for each isolate from the 2014 Danish population.

| **Isolate_ID** | **MLG** |
| --- | --- |
| 306 | 1 |
| 245 | 3 |
| 248 | 3 |
| 13-8-102 | 4 |
| 13-3-5 | 5 |
| 361 | 6 |
| 582 | 7 |
| 579 | 8 |
| 34 | 9 |
| 578 | 11 |
| 354 | 12 |
| 564 | 13 |
| 687 | 15 |
| 42 | 17 |
| 731 | 16 |
| 618 | 18 |
| 730 | 18 |
| 568.2 | 20 |
| 592 | 20 |
| 568.1 | 21 |
| 680 | 21 |
| 681 | 22 |
| 679 | 22 |
| 718 | 25 |
| 594 | 27 |
| 672 | 27 |
| 675 | 27 |
| 688 | 28 |
| 284 | 31 |
| 278 | 33 |
| 279 | 33 |
| 13-5-10 | 34 |
| 331 | 35 |
| 583 | 36 |
| 588 | 37 |
| 329 | 39 |
| 478 | 39 |
| 656 | 40 |
| 572 | 41 |
| 587 | 43 |
| 601 | 44 |
| 722 | 45 |
| 700 | 46 |
| 233 | 47 |
| 235 | 47 |
| 574 | 48 |
| 585 | 50 |
| 698 | 50 |
| 697 | 50 |
| 560 | 51 |
| 219 | 52 |
| 220 | 52 |
| 13-7-28 | 53 |
| 654 | 54 |
| 647 | 55 |
| 318 | 57 |
| 343 | 59 |
| 313 | 60 |
| 315 | 60 |
| 706 | 61 |
| 241 | 62 |
| 575 | 63 |
| 559 | 64 |
| 597 | 64 |
| 566 | 65 |
| 670 | 65 |
| 620 | 66 |
| 563 | 67 |
| 619 | 68 |
| 13-8-35 | 69 |
| 29 | 70 |
| 292 | 72 |
| 567 | 73 |
| 581 | 74 |
| 196 | 75 |
| 193 | 75 |
| 396 | 76 |
| 397 | 76 |
| 204 | 77 |
| 608 | 78 |
| 621 | 78 |
| 323 | 79 |
| 599 | 81 |
| 468 | 82 |
| 580 | 82 |
| 691 | 82 |
| 692 | 82 |
| 693 | 82 |
