# Supplementary material for: Population genomics of an outbreak of the potato late blight pathogen, Phytophthora infestans, reveals both clonality and high genotypic diversity
Source: Mol Plant Pathol. 2019 May 30;20(8):1134–46. doi: 10.1111/mpp.12819 (PMC6640178; doi:10.1111/mpp.12819)
Supplement: Supplementary file 8 — Table S3 Pairwise F ST values between sampling fields in 2014 based on both SSR and SNP datasets. [file MPP-20-1134-s008.docx]

**Table S3.** Pairwise-FST values between sampling fields in 2014 based on both SSR and SNP datasets.

| SSR |  |  |  |  |  |  |
| --- | --- | --- | --- | --- | --- | --- |
|  | M1 | M2 | M3 | M4 | M5 | M6 |
| M2 | 0.03234914 |  |  |  |  |  |
| M3 | 0.01483017 | 0.03219649 |  |  |  |  |
| M4 | 0.13213143 | 0.03883555 | 0.03396538 |  |  |  |
| M5 | 0.03187501 | 0.01601327 | 0.01476714 | 0.04764021 |  |  |
| M6 | 0.08593117 | 0.03967692 | 0.03709577 | 0.1026731 | 0.06099773 |  |
| M7 | 0.15980796 | 0.04817926 | 0.0421152 | 0.19881306 | 0.07489824 | 0.135905 |
|  |  |  |  |  |  |  |
| SNP |  |  |  |  |  |  |
|  | M3 | M4 | M5 | M6 |  |  |
| M4 | 0.00609207 |  |  |  |  |  |
| M5 | 0.00627864 | 0.011517865 |  |  |  |  |
| M6 | 0.0036232 | 0.008483203 | 0.01059787 |  |  |  |
| M7 | 0.00370848 | 0.009193433 | 0.009676529 | 0.0082084 |  |  |
|  |  |  |  |  |  |  |
