# Supplementary material for: Population genomics of an outbreak of the potato late blight pathogen, Phytophthora infestans, reveals both clonality and high genotypic diversity
Source: Mol Plant Pathol. 2019 May 30;20(8):1134–46. doi: 10.1111/mpp.12819 (PMC6640178; doi:10.1111/mpp.12819)
Supplement: Supplementary file 9 — Text S1 SSR analysis, whole genome and RAD‐seq library building. [file MPP-20-1134-s009.docx]

**Supplementary material, Montes et al. Population genomics of an outbreak of the potato late blight**.

*Materials &Methods*

*SSR Analysis*

DNA was extracted from axenic cultures of *P. infestans* by cutting a plug from the agar, slicing off the top layer where most of the mycelia were found, and placing it directly in QuickExtract™ Plant DNA extraction solution (Epicentre) and following the manufacturer's protocol. Of the twelve SSR loci and primers described by Y. Li *et al.* (2013a), only ten were used in the analysis: Pi02/PinfSSR3, PinfSSR11, PinfSSR8, PinfSSR4, Pi04, Pi70, PinfSSR6, Pi63, PinfSSR2, and Pi4B. Primers D13 and G11 resulted in either no peaks or too many peaks respectively in the majority of samples initially tested, and were therefore not included in the analyses. The dyes and primer concentrations used were kept the same as Li *et al.* (2013a). PCR was performed in a volume of 10 µl using VWR Red Taq 2x Master Mix, 2mM MgCl_2_, and varying concentrations of primers, following (Li *et al.,* 2013a). Amplifications were run in a BioRad T100™ Thermocycler (Bio-Rad Laboratories, Inc.) with initial denaturation at 95˚C for 15 minutes, followed by 30 cycles of 95˚C for 20s, 58˚C for 90s and 72˚C for 60s, and a final extension at 72˚C for 20 minutes. The amplified products were then diluted 1:10 with 8.75 uL HiDi™ Formamide and 0.25 µL GeneScan™ 500 LIZ™ dye size standard (Applied Biosystems) and run on an Applied Biosystems 3130XL Genetic Analyzer (Applied Biosystems). Multilocus gentoypes (MLGs) were identified using *GeneMapper v 5.0* (Applied Biosystems, 2005) and are listed in the supporting information (Table S1). In addition to the 83 new samples collected in 2014, microsatellite data from 84 samples collected in 2013 and previously analyzed by Montes *et al*. (2016) were included in some analyses.

*Whole Genome Sequencing*

DNA extraction was carried out on mycelium grown in a liquid pea medium, washed using Amoeba Saline solution (Page, 1988), and frozen at -20ºC until ready for use. The Qiagen DNeasy Plant Mini Kit and protocol was used for extraction. DNA was sheared into fragments of approximately 400 bp using a Diagenode Biorupter XL, followed by removal of small molecular weight fragments using the MinElute PCR purification kit. A 2% agarose gel was used to check for high molecular weight fragment size and integrity of DNA. Sheared DNA extracts were built into Illumina sequencing libraries using the NEBNext® DNA Library Preparation for Roche/454 kit, which is compatible with blunt-end adapters, following the manufacturer's protocol except for the use of MinElute PCR columns rather than a Streptavidin beads protocol. Blunt end adapters P5 and P7 were prepared according to Meyer & Kircher (2010). Initial DNA amplification was carried out through 12 cycles of PCR in 50 μL reactions using Phusion Hi-Fidelity PCR Master Mix, 0.2 μmol primer in PE 1.0, 0.2 μmol Illumina PCR reverse primer with unique indexing barcodes, and 5 μL of the DNA template. The PCR products were then purified using MinElute purification columns to remove enzymes and buffer. A BioAnalyser 2100 (Agilent) was used to determine insert sizes. Due to residual fragments longer than 1000 bp, a size-selection for 450 +/- 45 bp was performed using a LabChip XT (Caliper).

*Restriction-site Associated DNA sequencing (RAD-seq)*

A total of 78 samples were included in the RAD-seq study, including 58 individuals collected in Denmark in 2014, the five individuals isolated in 2013 for which whole-genome sequencing was also performed, two individuals from Mexico to serve as an outgroup, nine biological replicates, and four technical replicates (Table 1).

Cultures were grown in liquid pea medium in the dark at room temperature for 1-3 weeks, then harvested and freeze-dried overnight. The samples were crushed using a Qiagen TissueLyser with 3mm tungsten beads at 25 Hz for 2 minutes. A CTAB protocol was used for DNA extraction as follows: ground mycelia were mixed with 600 µl 2x CTAB and 0.2% ß-mercaptoethanol, incubated at 65˚C for 1 hr, followed by two treatments with 400 µl chloroform, a washing step with cold isopropanol, incubation at -20˚C, and two washing steps with cold 70% EtOH. The DNA was eluted in 50 µl AE buffer from the DNeasy Plant Mini Kit (Qiagen). The DNA extracts were then treated with 1 µl RNase (10 mg/ml), and cleaned using the Zymo Genomic Clean and Concentrator kit (Zymo Research) following the manufacturer's guidelines.

The concentration of DNA for each sample was quantified using a Qubit BR DNA kit (Thermo Fischer Scientist), to ensure that an equal amount (250 ng) of DNA was included per sample in the RAD-seq library preparation. Based on an in silico analysis using the program In SIlico Fingerprinting (Paris & Després, 2012), the restriction enzyme PstI was selected, and was estimated to generate 160 064 tags from the T30-4 reference genome (Haas et al., 2009).

The single-digest RAD libraries were prepared according to the original protocol of Baird et al. (2012), with some modifications for fungal DNA as adapted in Maurice et al. (in prep.) Briefly, after restriction of the genomic DNA, the samples were uniquely ligated with P1 adapters, pooled, and then sheared by sonication using a 2100 Bioruptor (Diagenode) with 9 cycles of 30s ON and 30s OFF. The random shearing of the DNA ensures that all restriction sites in the genome can potentially be included by eliminating any length bias in amplification steps, and also aids in the easy identification and removal of PCR clones farther down the line (Davey et al., 2011). Libraries were then concentrated using a MinElute Gel Extraction Kit (Qiagen), and run on a Blue Pippin Size Selector (Sage Sciences) with a 2% DF gel cassette to retain fragments between 350-650 bp long. Libraries were then purified using AMPure beads (Agencourt), and P2 adapters were ligated using T4 ligase (NEB).

The six prepared libraries were amplified in 25 µl reaction volumes, and the PCR products were cleaned with AMPure beads. Libraries were quantified and pooled into two Illumina HiSeq lanes. Both biological and technical replicates were prepared and sequenced in separate libraries.

The RAD libraries were sequenced using 2 x 250bp paired-end sequencing on the Illumina HiSeq 4000 platform, performed at the Norwegian Sequencing Centre ([www.sequencing.uio.no](#_blank)).
